# Design and acoustic characterization of a test bench for aeroacoustic studies under controlled turbulent flow

**DOI:** 10.1016/j.mex.2026.103803

**Published:** 2026-01-23

**Authors:** Pablo Gianoli Kovar, José Cataldo Ottieri

**Affiliations:** Fluid Mechanics and Environmental Engineering Institute, School of Engineering, Universidad de la República, Montevideo, Uruguay

**Keywords:** Aeroacoustic characterization, Turbulent flow acoustics, Wind–structure interaction, Residual energy spectrum

## Abstract

Aeroacoustic phenomena generated by wind–structure interaction have gained increasing relevance due to the widespread use of lightweight and permeable façade elements. Several studies have shown that such configurations may emit tonal noise, yet their prediction and mitigation remain challenging. To address this, a dedicated experimental facility was developed to study, under controlled conditions, the coupling between the flow field and the acoustic field.

The test bench was designed following aerodynamic similarity criteria and theoretical references on turbulent jets. Material selection was supported by absorption and transmission-loss measurements using a Kundt tube according to ISO 10534-2:1998 (Reaffirmed 2021) [1], while the acoustic characterization of the facility was conducted following ISO 3744:2010 [2]. This article presents a validated and reproducible experimental methodology intended to support future investigations of wind-induced noise in architectural and structural components.

• Design of a controlled aeroacoustic test bench verified through standardized acoustic measurements.
• Implementation of a multi-stage characterization protocol: material testing, background-noise mapping, and vibration analysis.
• A reproducible framework for flow–acoustic coupling experiments on façade elements under controlled turbulent flow.

## Graphical abstract

**Figure:**
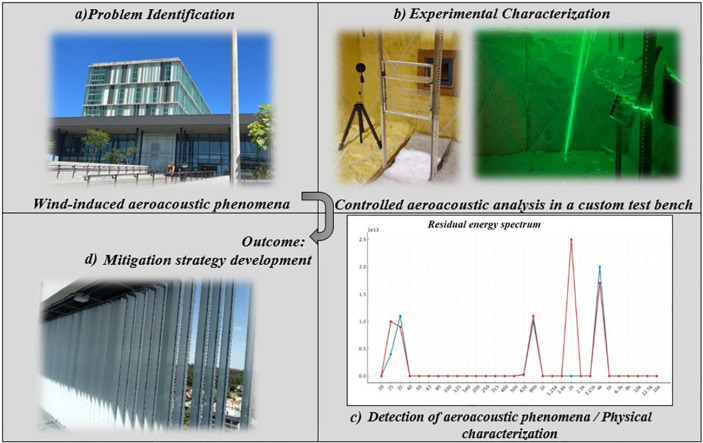

## Specifications table

**Table:** 

**Subject area**	Environmental Science
**More specific subject area**	Aeroacoustics, wind–structure interaction, experimental methods
**Name of your method**	Design and acoustic characterization of a controlled-turbulence test bench for aeroacoustics studies
**Name and reference of original method**	Not applicable
**Resource availability**	Not applicable

## Value and applicability of the proposed method

The experimental methodology and test bench presented in this work are intended for researchers and professionals involved in the study of wind-induced noise and flow–structure interaction phenomena. Potential users include aeroacoustics and wind engineering research groups, building and environmental acoustics laboratories, façade engineers, architectural designers, and acoustic consultants engaged in the assessment and mitigation of wind-related noise in the built environment. The method is also relevant for public agencies and regulatory bodies concerned with environmental noise control and urban acoustic comfort.

The main value of the proposed method lies in providing a controlled and reproducible experimental framework for investigating aeroacoustic phenomena that are difficult to isolate through field measurements or purely numerical approaches. By decoupling flow-induced acoustic emissions from background noise, structural vibrations, and uncontrolled boundary conditions, the test bench enables reliable identification of tonal and broadband components generated by specific geometries under well-defined flow conditions.

This approach is particularly useful for the early-stage evaluation of lightweight and permeable architectural elements—such as shading devices, slats, perforated panels, and façade assemblies—where small geometric variations can lead to significant acoustic effects. The documented method supports comparative and parametric studies, facilitates inter-laboratory reproducibility, and provides experimental evidence to complement numerical modeling and design-based mitigation strategies for wind-induced aeroacoustic problems.

## Background

Wind-induced aeroacoustic phenomena have gained increasing relevance in recent years due to the growing use of lightweight, permeable, and geometrically complex architectural elements in façades and structural systems. Numerous documented cases, both nationally and internationally, show that common building components—such as slats, shading devices, perforated plates, joints, and repeating façade assemblies—can generate tonal noise when subjected to atmospheric turbulence. Although these emissions may not compromise structural performance, they can significantly affect urban acoustic comfort and the perception of the built environment. Well-known international examples include the Golden Gate Bridge in San Francisco, where the installation of new guardrails in 2020 led to intense tonal noise due to wind–structure interaction, requiring subsequent mitigation measures (BBC News, 2020). At a regional and local scale, similar aeroacoustic phenomena affecting buildings and façade elements have been documented and analyzed in detail in previous work by the author [3], including case studies in Uruguay (the World Trade Center complex in Montevideo, 2012) and Argentina (commercial tower developments in the Núñez district, Buenos Aires, 2025). These cases highlight the growing relevance of wind-induced aeroacoustic effects in contemporary architectural design and urban developments in the region.

Despite the global interest in wind–structure interaction, the scientific literature addressing aeroacoustic behavior in architectural applications remains comparatively limited. Previous work, including the author’s master degree’s thesis [3], collected and analyzed multiple instances of wind-induced tonal noise in real buildings. Some configurations exhibited clearly identifiable aeroacoustic generation mechanisms, while in other cases the origin of the tonal radiation could not be determined with confidence. These findings highlight an existing methodological gap: the lack of experimental facilities specifically designed to reproduce and study wind-induced tonal phenomena under controlled conditions.

The theoretical foundation of this work is Lighthill’s aeroacoustic analogy [4], which reformulates the nonlinear Navier–Stokes equations into an inhomogeneous wave equation with source terms related to turbulence and vorticity. This formulation was later extended by Curle to account explicitly for the influence of rigid bodies immersed in the flow, making it particularly relevant for flow–structure interaction problems [5]. While this framework has been extensively applied in fields such as automotive engineering, aerospace acoustics, and wind-turbine noise, its application to building-related aeroacoustic phenomena remains comparatively limited. In practice, the strong sensitivity of aeroacoustic emissions to geometric details, boundary conditions, and flow characteristics makes controlled experimentation essential for understanding and predicting sound generation mechanisms in building elements.

Given these limitations, the development of a dedicated experimental method was required—one capable of isolating the aeroacoustic interaction between a controlled turbulent flow and architectural elements, while allowing precise measurement of both the acoustic field and the flow field. The controlled-turbulence aeroacoustic test bench presented in this article was conceived as a technical response to this need. It provides a reproducible laboratory environment with adjustable geometry, acoustic isolation, and reduced background noise, enabling systematic investigation of wind-induced tonal phenomena in building-related components.

## Method details

The proposed methodology follows a structured sequence consisting of: (i) design and verification of the aeroacoustic test bench, (ii) material and structural characterization, (iii) quantitative characterization of the turbulent flow, (iv) acoustic baseline definition, and (v) application of the residual energy approach to detect flow-induced aeroacoustic phenomena.

### Verification procedures in the test bench

According to ASCE Manual 67 [6], the design of experimental benches or wind tunnels requires a series of fundamental verifications to ensure an appropriate representation of the physical problem under study. In the present work, the verifications addressed both the fluid-dynamic behavior of the flow and the acoustic conditions of the installation. These checks include the selection of the test bench scale, blockage considerations in the test section, velocity-scale and Reynolds-number considerations, all of which are detailed below.

### Selection of the test bench scale

The geometric scale of the model must preserve the similarity between the dimensions of the prototype and the characteristics of the atmospheric flow, according to the following relationships [1]:

$${\left(\frac{L_{b}}{z_{0}}\right)}_{m}={\left(\frac{L_{b}}{z_{0}}\right)}_{p}$$

$${\left(\frac{L_{b}}{z_{g}}\right)}_{m}={\left(\frac{L_{b}}{z_{g}}\right)}_{p}$$

$${\left(\frac{L_{b}}{L_{t}}\right)}_{m}={\left(\frac{L_{b}}{L_{t}}\right)}_{p}$$

where:

• $L_{b}$: characteristic dimension of the building or structural element
• $z_{0}$: aerodynamic roughness length of the terrain
• $z_{g}:$ height of the boundary layer or of the significant flow region
• $L_{t}$: turbulence length scale

For detailed aeroacoustic studies, turbulence simulation may be partial, provided that the kinetic energy of the small scales is properly represented. In the present test bench, the model and the prototype share the same geometric scale. This geometric scale can be equal to one when real plates or profiles typically used in building façades or metallic structures are employed, or smaller than one in the case of slats or sectional configurations such as those used by [7] and [8] .

### Blockage considerations

Minimizing blockage effects in the test section is essential when selecting the model scale. Blockage occurs when the size of the model becomes significant relative to the cross-sectional area of the tunnel, producing artificial flow accelerations and distortions around the model. The blockage-induced flow acceleration can be quantified by measuring the wind speed in the model plane, whereas flow distortions are more difficult to correct.

According to [6], when the blockage ratio—defined as the ratio between the frontal area of the model and the cross-sectional area of the wind tunnel—is below 5%, distortion effects are negligible, and a correction for the local increase in flow velocity is generally sufficient. For blockage ratios between 5% and 10%, distortion effects become significant and should be accounted for through complementary analyses or empirical corrections. Above 10%, the validity of the experimental data must be verified through tests at smaller scales or with alternative models that reduce interference in the flow.

### Velocity-scale and Reynolds number considerations

The selection of the reference wind speed in the test bench depends on the phenomenon under investigation. In studies focused on flow characterization—such as pedestrian-level wind assessment or the evaluation of aerodynamic forces on buildings and structures—the choice of the velocity scale may be relatively arbitrary, provided that aerodynamic similarity between the model and the prototype is maintained (i.e., Reynolds-number independence).

In practice, achieving strict similarity between the atmospheric flow and the flow reproduced in the test bench is challenging, since it is not always possible to scale both the mean velocity and the turbulent Reynolds number simultaneously. Reynolds-number independence in aerodynamic flows occurs for ratios $\frac{u^{*}z_{o}}{\upsilon }>2.5$, where $u^{*}$ is the friction velocity, defined as (τ_o_/ρ)^1/2^; τ_o_ ​ is the surface shear stress; ρ is the air density; and z_0_​ is the aerodynamic roughness length.

When the model Reynolds number

$$Re_{m}=\frac{V_{h}L_{m}}{\upsilon }$$

(Where $V_{h}$ is the reference wind speed and $L_{m}$ is the characteristic model dimension) falls below a critical value, flow corrections or surface-roughness modifications may be required to improve local similarity. For models with sharp edges, achieving a minimum $Re_{m}$ is essential in order to reduce local viscous effects.

It should be noted that the frequency range of turbulence spectra also depends on the Reynolds number. In general, flow distortion and variations in pressure distributions tend to be negligible for $Re_{m}$ values greater than 10⁴. However, caution is required when dealing with slender or surface‐mounted elements aligned with the flow, since local viscous effects, boundary-layer transitions, or laminar separation bubbles may still occur even at moderate Reynolds numbers. When the Reynolds number of the model does not satisfy the independence condition, its consequences can be assessed through comparisons with full-scale data, the use of larger-scale models, or by repeating the tests at different reference wind speeds $V_{h}$.

### Characterization of the turbulent flow

To support the assumption of controlled turbulent flow within the test bench, a quantitative characterization of the flow field was performed using hot-wire anemometry. Point-wise velocity measurements were carried out at representative locations downstream of the diffuser, including the region where the test samples are installed.

The hot-wire measurements provided the mean velocity *U* and the standard deviation of the fluctuating component σu, allowing the turbulence intensity I=σ_u_/U to be quantified for the different operating conditions. Measurements were conducted for both the minimum- and maximum-velocity blower settings used throughout the acoustic characterization.

At a distance of 1 m from the diffuser outlet, corresponding to the typical position of a 0.20 × 0.20 m test sample, the mean incident velocities were approximately 9 m/s and 12 m/s for the minimum- and maximum-velocity conditions, respectively (Table 5). The measured velocity fluctuations resulted in turbulence intensities within the range expected for shear-dominated turbulent jets downstream of a diffuser, confirming that the flow is fully turbulent and repeatable under the selected operating conditions. Spatial variations of the velocity field were further assessed by mapping the velocity contours in the measurement plane (Fig. 3). The footprint of the test sample lies within the region bounded by the 1.10·Ū isoline, indicating that local velocity deviations remain within ±10% of the plane-averaged value. Although larger gradients exist across the full measurement plane, this level of non-uniformity is consistent with atmospheric-like turbulent shear flows and is acceptable for the present aeroacoustic experiments, provided that the local flow conditions are well characterized.

Overall, the combination of mean velocity levels, measured velocity fluctuations, and spatial repeatability demonstrates that the test bench produces a controlled turbulent flow suitable for systematic aeroacoustic investigations of wind–structure interaction phenomena.

**Fig. 1:**
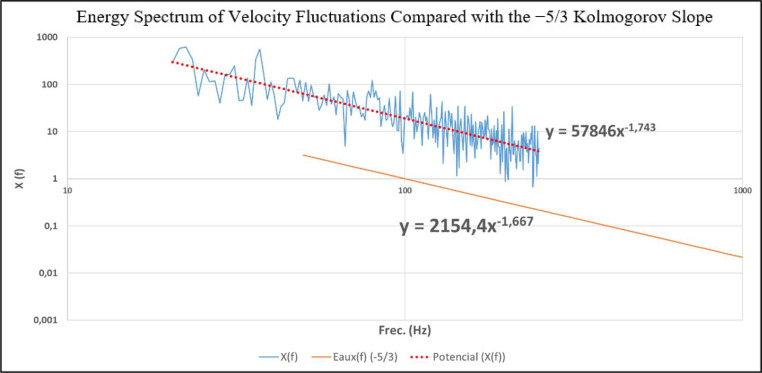
Velocity fluctuation energy spectrum obtained from hot-wire measurements at a representative point within the measurement grid (75 cm downstream of the diffuser). The dashed line indicates a −5/3 slope, shown as a reference for the inertial subrange of fully developed turbulent flows.

In addition, the spectral content of the velocity fluctuations was evaluated at a representative point within the hot-wire measurement grid. Fig. 1 shows the velocity energy spectrum together with a reference −5/3 slope. Over a finite frequency range, the measured spectrum exhibits a decay consistent with the inertial subrange expected in fully developed turbulent flows. This result provides further quantitative support that the generated flow shares key physical characteristics with wind-driven turbulent shear flows relevant for aeroacoustic investigations, without implying strict atmospheric similarity.

### Acoustic considerations

Standing waves may form in various ways inside an enclosed space. The simplest case occurs when a low-frequency sound wave resonates between two opposing surfaces, producing a constant-amplitude reinforcement at that frequency due to constructive interference. This phenomenon is associated with the axial modal components of the room, which appear at frequencies whose wavelengths are equal to twice the distance between the reflective surfaces. The lowest resonance frequency of an enclosure corresponds to the condition in which the wavelength equals twice its largest dimension. In this situation, the standing wave is independent of time and depends only on the spatial position, exhibiting the same amplitude at a fixed point in space at any instant.

The modal distribution of a rectangular enclosure can be obtained from the classical expression described by [9], and widely summarized in standard acoustic references such as [10], which is valid for three-dimensional resonant systems with idealized rigid boundaries:

(1) $$f\left(n_{x},n_{y},n_{z}\right)=\frac{c_{0}}{2}\sqrt{{\left(\frac{n_{x}}{l_{x}}\right)}^{2}+{\left(\frac{n_{y}}{l_{y}}\right)}^{2}+{\left(\frac{n_{z}}{l_{z}}\right)}^{2}}$$

where:

• $c_{0}$ is the speed of sound in air (m/s).
• $n_{x}$, $n_{y}$, $n_{z}\;$are integer mode orders.
• $l_{x}$, $l_{y}$, $l_{z}$ are the dimensions of the enclosure.

Depending on the combination of dimensions involved, three types of room modes can be distinguished:

• Axial modes: involve only two opposing surfaces (e.g., floor and ceiling).
• Tangential modes: involve the interaction of four surfaces.
• Oblique modes: involve six or more surfaces.

The relative strength of these modes depends on the number of surfaces participating in the resonance. For an equal excitation power, tangential modes exhibit sound pressure levels approximately 3 dB lower than axial modes, while oblique modes are typically 6 dB lower. These differences arise because to reach the same amplitude as an axial mode, a tangential mode would require roughly twice the acoustic power, and an oblique mode about four times the power ([9] and [10]).

A common strategy to mitigate the effects of room modes is to increase the damping factor by incorporating sound-absorbing materials that are effective at low frequencies. This reduces both the maximum amplitude of the resonances and the bandwidth over which they occur. A more targeted treatment consists of identifying the problematic modal frequencies and adding materials with high absorption in those specific bands, thereby optimizing the acoustic response of the test bench.

Another relevant parameter is the reverberation time (T_R_), defined as the time required for the acoustic energy in an enclosure to decay to one millionth of its initial value after the sound source has stopped. The reverberation time can be estimated using the Eyring–Norris equation ([9] and [13]), which is valid for enclosures with a diffuse sound field distribution:

(2) $$T_{R}=0.161\frac{V}{4\gamma V-S_{T}Ln\left(1-\alpha_{T}\right)\;}$$

where:

• γ is the air-absorption coefficient, which depends on relative humidity and ambient temperature.
• V is the volume of the enclosure (m^3^).
• $S_{T}$ is the total surface area of the enclosure (m^2^).
• $\alpha_{T}$ is the average absorption coefficient of the enclosure.
  (3) $$\alpha_{T}=\frac{\sum \alpha_{i}S_{i}}{S_{T}}$$

Since the air-attenuation constant (γ) is small, the term 4γV becomes relevant only at frequencies above 2000 Hz and in large-volume enclosures. In the case of the test bench designed here, this term is negligible due to the relatively small volume of the test section.

### Test bench design

The test bench constructed is equipped with a Toro PowerJet F700 blower. This device allows a wide range of flow velocities to be achieved, covering conditions equivalent to atmospheric wind speeds at which aeroacoustic phenomena have been reported in both field measurements and laboratory experiments. The overall structure consists of a two-stage diffuser, composed of a rectangular section tube followed by a truncated-pyramidal cone, which exhausts into an insulated chamber where the test samples are mounted. The diffuser was designed to operate without boundary-layer separation, thereby avoiding a free-jet regime and ensuring a controlled flow over the obstacle. Inside the test chamber, a set of microphones is placed at predefined positions to record the acoustic pressure fluctuations.

The chamber was built using ¾'' (18 mm) wooden panels, internally lined with 50 mm mineral wool covered on one side with an aluminum foil. In addition, two transparent acrylic windows measuring 600 mm × 300 mm were installed to allow direct observation of the flow–obstacle interaction.

### Dimensions and design considerations

**Fig. 2:**
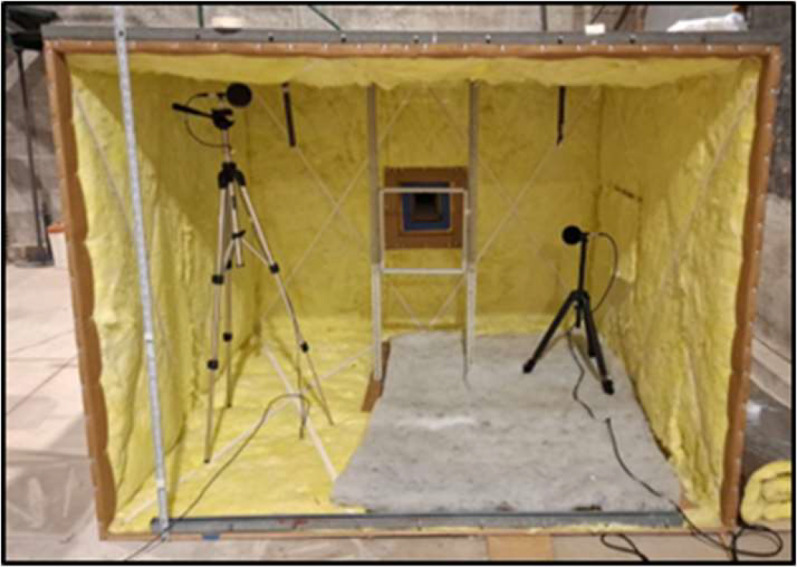
Custom-built test bench used for the experimental aeroacoustic characterization of wind–structure interaction phenomena. The facility allows controlled airflow conditions and repeatable measurement configurations for the evaluation of sound generation mechanisms associated with permeable and lightweight façade elements.

The test bench was designed to accommodate up to 400 mm × 400 mm, with different geometries. Based on these samples and the recommendations found in the literature, the working volume of the bench was defined as 6 m³, corresponding to a chamber measuring 2.0 m × 2.0 m × 1.5 m (see Fig. 2). Construction was carried out using 2.20 m × 1.50 m wooden panels, ensuring adequate structural rigidity and acoustic insulation.

During the design stage, the following considerations were taken into account:

• **Location of the test bench:** the system had to be installed in a controlled environment, isolated from external noise sources. For this reason, a large and quiet room at IMFIA was selected, where the Institute’s wind tunnel is located. This space provides low background noise conditions and allows comparisons with previous physical modelling studies of atmospheric wind.
• **Baseline noise determination:** once the system was installed, an acoustic baseline for the chamber (reference sound level without an obstacle) was established. This baseline makes it possible to distinguish the intrinsic noise of the blower and the room from the aeroacoustic phenomenon of interest. This characterization is essential for the subsequent analysis using the subtraction method developed as part of this work.
• **Operational workspace:** in addition, to meet the required aerodynamic and acoustic criteria, the test bench needed to provide sufficient working space for the installation and handling the samples, as well as for placing measurement instruments without disturbing the flow field.
• **Velocities and Reynolds number:** the Toro PowerJet F700 blower can reach flow velocities of up to 60 m/s at its outlet nozzle (3½’’ duct). The flow is directed toward the two-stage diffuser, which distributes the stream uniformly within the test chamber. According to the reference literature ([3]), these operating conditions allow achieving Reynolds numbers on the order of 10⁴. However, the exact Reynolds number depends on the characteristic length of the element being tested, so the effective Re may vary depending on the size and geometry of each specimen. This range is nonetheless appropriate for representing the flow regime around the architectural components investigated in this study.
• **Blockage effect:** the ratio between the frontal area of the model and the cross-sectional area of the test section was verified to remain below 5%, in order to minimize interference with the chamber walls. For samples measuring 200 mm × 200 mm (0.04 m²) and an effective test-section area of 3 m², the resulting blockage ratio is 1.3%, a value considered acceptable in the technical literature ([6] In this context, even samples up to 400 mm × 400 mm (0.16 m²) can be tested without exceeding the 5% threshold associated with critical distortion. As an additional verification, point-wise velocity measurements were carried out using hot-wire anemometry in the test region, verifying that the flow was not significantly affected within the blockage ranges considered.

**Fig. 3:**
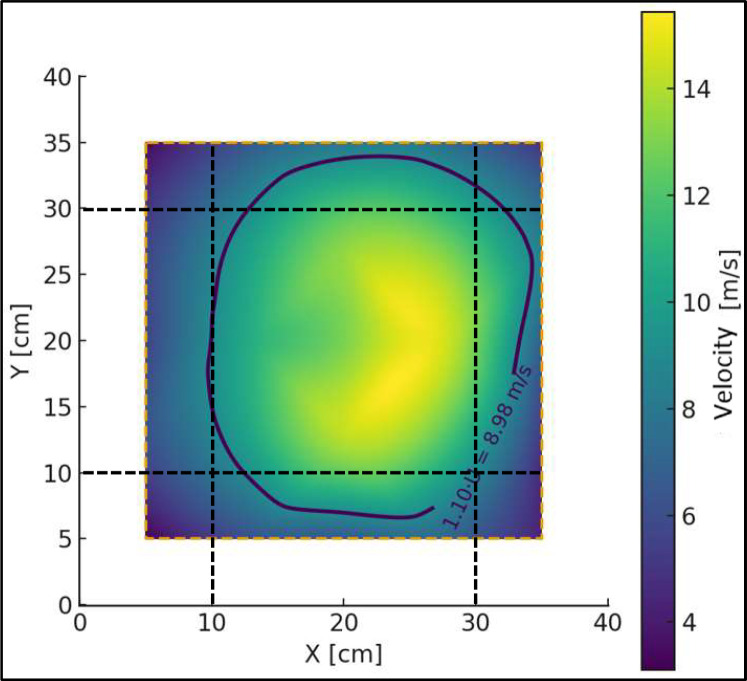
Velocity contour at 1 m from the diffuser for the maximum-speed operating condition. The dashed square indicates the 0.20 × 0.20 m area where the samples are mounted during the acoustic tests. The black contour corresponds to 1.10·Ū, marking the region where the local velocity deviates by less than approximately +10% from the plane-averaged velocity Ū, indicating that the tested sample is exposed to flow conditions with limited velocity deviations, suitable for repeatable aeroacoustic measurements.

To evaluate the effective uniformity of the flow reaching the test samples, the velocity field at 1 m from the diffuser was analyzed by superimposing the footprint of a 0.20 × 0.20 m specimen and the isoline corresponding to 1.10·Ū, where Ū is the mean velocity in the measurement plane (Fig. 3). This isoline marks the boundary where the local velocity exceeds the mean value by 10% and is used as a reference to assess regions with limited velocity deviation. The footprint of the 0.20 × 0.20 m specimen lies entirely within this region, indicating that the flow impinging on the sample exhibits limited velocity deviations within the region of interest. Although larger gradients exist across the full 0.30 × 0.30 m measurement plane, such non-uniformity is consistent with the shear typically observed in atmospheric flows around façade elements and is acceptable for the present aeroacoustic experiments, provided that the local flow conditions are well characterized and repeatable. The objective is not to achieve global flow uniformity, but to ensure controlled and representative flow conditions at the specimen location. It should be noted that the velocity range within the sample footprint reflects the inherent shear of the flow and does not imply strict uniformity. The observed velocity variations are intentionally preserved to represent atmospheric wind conditions acting on façade elements, rather than an idealized uniform inflow.

• **Natural frequencies of the chamber:** To avoid coincidences between the modal frequencies of the chamber and the frequency bands in which aeroacoustic phenomena are expected to appear, the natural frequencies were estimated assuming that one side of the chamber (lx) remains open to the exterior, corresponding to the flow outlet. Under this assumption, the modal analysis was restricted to the y and z directions. The results indicate that the lowest natural frequency of the chamber occurs at 86 Hz, corresponding to the axial mode in the y-direction, while the first axial mode in the z-direction appears at 114 Hz. These frequencies are significantly lower than the bands in which the aeroacoustic interaction between the flow and the obstacle is expected to occur (typically above 500 Hz), thereby minimizing the risk of modal interference between chamber resonances and the aeroacoustic emissions of interest.

### Reverberation time

The reverberation time (T_R_) can play a significant role during the recording of the aeroacoustic phenomenon, as it determines the temporal persistence of sound energy within the enclosure. Although the test chamber has a relatively small volume (≈6 m³) and one of its sides remains open to the exterior, it was considered appropriate to determine this parameter in order to avoid potential acoustic interferences that could affect the measurements. The reverberation time was estimated as an internal check to verify low acoustic persistence within the test bench; the enclosure is not intended to be treated as a room in the sense of standardized room acoustics methods.

**Fig. 4:**
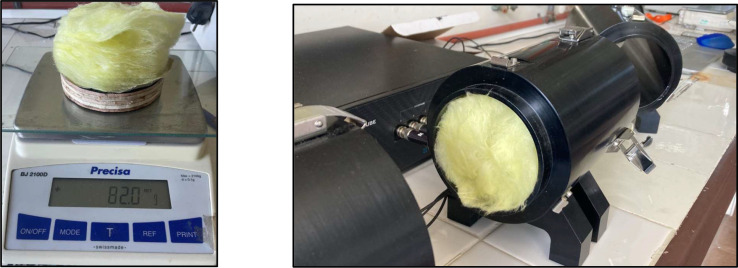
Tested sample of the proposed material and acoustic characterization performed in an impedance tube. The impedance tube measurements provide reference acoustic properties of the material prior to its evaluation under flow-induced aeroacoustic conditions in the test bench.

To estimate T_R_, an acoustic analysis of the construction materials of the test bench was first carried out. As described in previous sections, the chamber was built using ¾’’ wood panels, internally lined with 50 mm mineral wool and a thin aluminium foil layer (Fig. 4).

The composite material (wood + mineral wool + aluminium) was tested using a Holmarc® ITA-219 impedance tube belonging to the Acoustics Laboratory of the Environmental Engineering Department at IMFIA (Fig. 3). This device, manufactured from anodized aluminum, allows the determination of acoustic absorption coefficients and transmission loss in accordance with [1], which is based on the transfer-function method. This method separates the incident and reflected components of the sound wave, enabling the absorption curve of the material under analysis to be obtained.

**Fig. 5:**
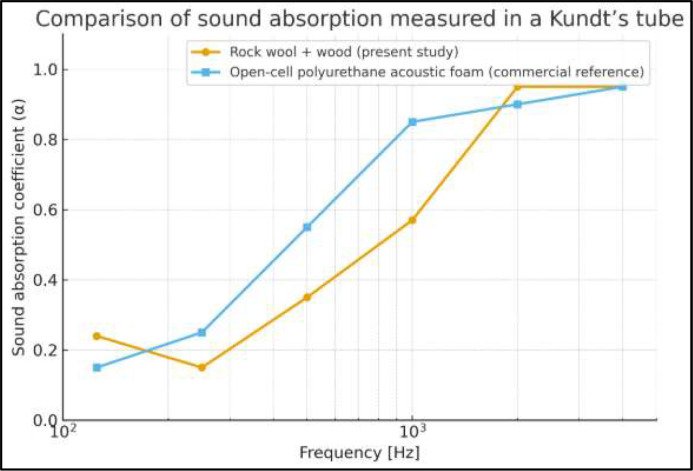
Normal-incidence sound absorption coefficients (α) of the composite material compared with a commercial reference material, obtained from impedance tube measurements. The comparison provides a baseline acoustic characterization of the materials prior to their evaluation under flow-induced aeroacoustic conditions in the test bench.

Based on the experimental measurements, the octave-band absorption curve of the composite material was obtained. Fig. 5 shows such result and the curve of a commercial reference material.

The composite material rock wool and wood absorption coefficients, measured in the Kundt tube, exhibits a qualitatively similar behaviour to the commercial polyurethane acoustic foam one, and also quantitatively for frequencies below 200 Hz and above 1 kHz. In the mid- and high-frequency bands the material shows high absorption, as shown in Table 1. Although its performance at medium and low frequencies is slightly lower—due to the stiffness of the wood, which limits wave penetration—the overall results indicate that the proposed material achieves a good acoustic performance, making it suitable for use in the construction of the test bench and in future experimental applications.

**Table 1:** Absorption coefficients (α) of the composite material compared with a commercial reference material.

Frequency (Hz)	Polyurethane acoustic foam (commercial)	Rock wool + wood
**125**	0.15	0.24
**250**	0.25	0.15
**500**	0.55	0.35
**1000**	0.85	0.57
**2000**	0.90	0.95
**4000**	0.95	0.95

By applying Eq. 2, based on the Eyring–Norris formulation [12], together with the average absorption coefficients obtained, the reverberation time was estimated in octave bands. The results are summarized in Table 2.

**Table 2:** T_R_ by Octave Bands.

Octave Band (Hz)	125	250	500	1000	2000	4000
Average absorption coefficient (αₜ)	0.20	0.13	0.30	0.48	0.81	0.81
R_T_ (s)	0.20	0.30	0.10	0.10	0.03	0.03

The results confirm that the chamber exhibits very low reverberation times (≤ 0.3 s) across the entire frequency range analysed, with a clear decrease toward the higher frequencies. This behaviour ensures that the recorded acoustic signals originate primarily from the flow–obstacle interaction, without significant resonance or reflection effects within the chamber. Overall, the selected composite material provides an acoustically damped and controlled environment, enabling reliable detection of pressure fluctuations and energy peaks associated with the vortices generated during the aeroacoustic tests.

### Complementary transmission loss assessment

**Fig. 6:**
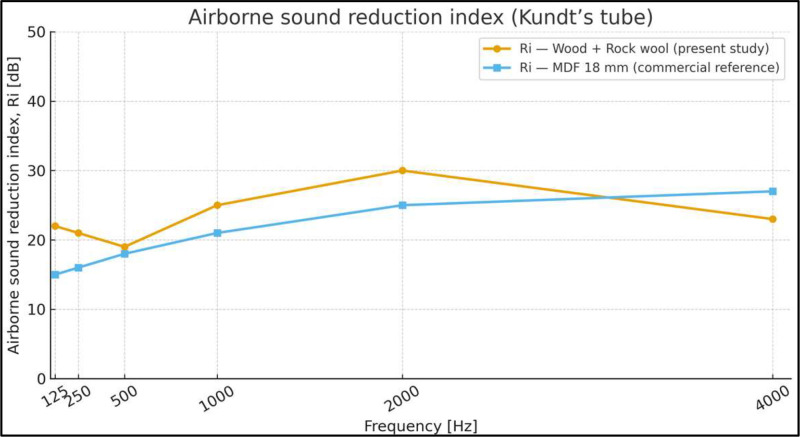
Normal-incidence sound reduction index (Rᵢ) of the proposed material, obtained from impedance tube measurements. This characterization provides complementary acoustic information of the material prior to its evaluation under controlled flow-induced aeroacoustic conditions in the test bench.

Fig. 6 presents the sound transmission loss experimentally determined using the impedance tube. The results show that the composite material wood - rock wool exhibits a behaviour similar to an 18-mm MDF panel across the analysed frequency range. According to the obtained results, the proposed material would provide an acoustic transmission loss on the order of 30 dBA between the interior and exterior of the test bench.

The sound reduction index (Rᵢ), which quantifies the airborne sound insulation of a panel as the logarithmic ratio between incident and transmitted sound energy, was obtained from three independent repetitions, calculating the average in the energy domain (i.e., averaging $10^{0.1*Li}$​), as required for levels expressed in decibels. For reference purposes only, the experimental transmission loss values were compared with theoretical estimates based on the Mass Law [11].

The physical properties of the composite material used for the transmission loss assessment, including thickness, density, and surface mass, are summarized in Table 3. The corresponding sound reduction indices obtained experimentally and their comparison with theoretical estimates are reported by octave band in Table 4.

**Table 3:** Material properties.

Sample	Weight (g)	Thickness (mm)	Volume (cm³)	Density (g/cm³)	Surface mass (kg/m²)
**Proposed material**	82.0	68.0	71.0	1.2	81.6

**Table 4:** Sound reduction indices by octave bands.

Frequency (Hz)	Wool + Wood (experimental)	Rock wool + Wood (theoretical)
**125**	22	25
**250**	21	31
**500**	20	37
**1000**	25	43
**2000**	30	49
**4000**	23	55

Overall, the absorption, reverberation time, and transmission loss measurements were conducted to verify that the test bench provides an acoustically controlled environment suitable for aeroacoustic experiments. The absorption and reverberation time results confirm that internal reflections are effectively suppressed, while the transmission loss measurements ensure adequate acoustic decoupling between the interior of the test bench and the surrounding environment. These characterizations are not intended as a comprehensive study of material acoustic performance, but as supporting steps within the methodological framework to guarantee reliable detection of flow-induced pressure fluctuations and tonal components.

### Test bench characteristics

**Fig. 7:**
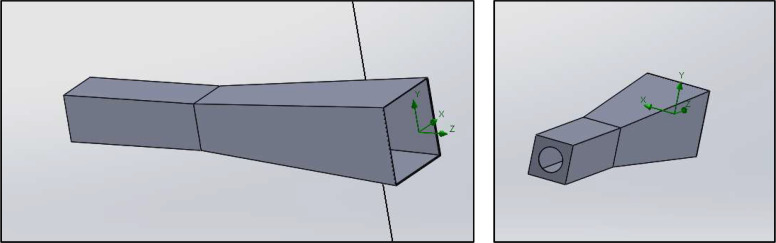
Custom-designed diffuser used in the test bench to condition the airflow prior to its entrance into the measurement chamber. The geometry was developed using CAD modeling (SolidWorks) to ensure controlled and reproducible flow conditions; detailed dimensions are provided in the text.

The diffuser consists of two consecutive sections (Fig. 7):

• **First section:** a straight square duct measuring 100 mm × 100 mm and 500 mm long. This section ensures the development of the flow and allows the installation of a differential micromanometer to measure the gauge pressure under different operating conditions. These measurements are used to relate the gauge pressure to the mean velocity inside the bench, providing an essential tool for calibration prior to each test. This configuration is consistent with the classical behavior of turbulent jets described by [14] where the potential core collapses and the shear layer transitions to fully developed turbulence prior to geometric expansion. Ensuring this condition reduces the risk of flow separation inside the diffuser and promotes a more uniform velocity field entering the test chamber.
• **Second section:** a truncated-pyramidal diffuser (500 mm long) designed to expand and uniformly distribute the flow toward the test chamber. Combined with the upstream straight duct (500 mm), the total diffuser length is 1.0 m. The diffuser assembly is mounted at the center of the inlet face of the test bench, aligned with the main flow axis.

The above dimensions fully define the diffuser geometry and its integration within the test bench, ensuring reproducibility of the flow-conditioning stage.

**Fig. 8:**
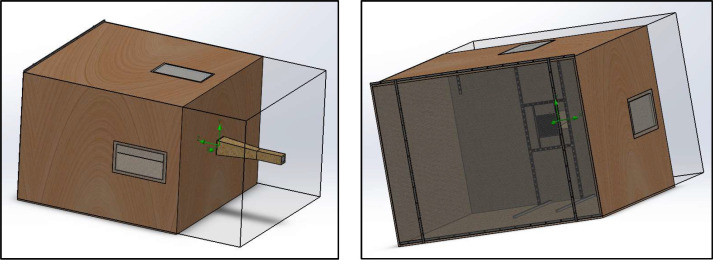
Central chamber and working area of the experimental test bench, where the aeroacoustic measurements are performed. The geometry was developed using CAD modeling (SolidWorks) to define the measurement volume and experimental layout.

The main body of the test bench (Fig. 8) includes a test section 2.0 m high, 1.50 m wide, 2.0 m long and an opening at the opposite end for flow exhaust. This configuration is intended to maintain a steady flow field, with uniform velocities and minimal interference from the walls.

**Fig. 9:**
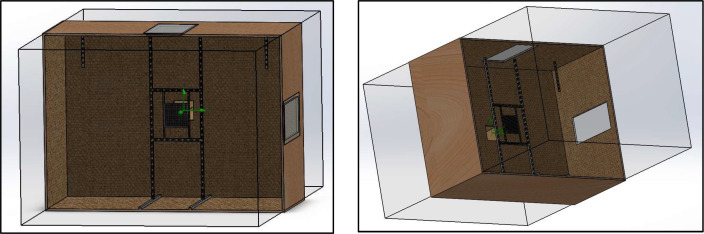
Location of the element under test within the central working area of the experimental test bench. The position is defined to ensure reproducible flow conditions and consistent aeroacoustic measurements, based on the CAD model developed in SolidWorks.

Fig. 9 illustrates a typical perforated plate used as a sample, with dimensions of 200 mm × 200 mm supported on a frame. This support frame is adjustable, enabling the distance between the sample and the diffuser outlet to be modified within a useful range of 1.20 m, which is essential for exploring different flow-development conditions and impact velocities. Additionally, the frame includes a graduated semicircle that allows the inclination angle of the sample to be varied with respect to the flow axis, representing different wind attack angles. This versatility will allow future stages of the study to analyse the influence of sample orientation on the generation and propagation of aeroacoustic noise.

### Acoustic verification of the test bench

#### Introduction and microphone installation

For the experimental analysis, three Class-1 precision sound level meters compliant with IEC 61672-1:2013 [15] and [16] were used: two Brüel & Kjær 2250 devices and one Casella CEL 63-C. All devices allow real-time third-octave-band measurements under different frequency weightings (A, C, and Z), with fast, slow, and impulse time responses. The Brüel & Kjær units record second-by-second time histories in third-octave bands, while the Casella stores one-minute averages and retains A-weighted values at a one-second resolution. Each sound level meter is equipped with an extension cable that enables the microphone to be placed inside the test bench while the instrument body remains outside, allowing real-time monitoring. Additionally, six Pulsar NoisePen dosimeters were used, capable of recording unweighted sound pressure levels every second. These devices were employed as a complementary resource to map the pressure field inside the bench under different experimental configurations.

Microphone positioning is a critical aspect as improper placement can generate errors due to direct flow incidence or sensor overloading. Since standard microphones tolerate wind speeds below 5 m/s when fitted with a windscreen, efforts were made to position them in shielded areas or in locations protected from direct airflow. Although no universal reference layout exists for aeroacoustic test benches, the initial microphone arrangement was based on the work [16], who analysed trailing-edge noise in flat plates immersed in turbulent flow. According to their methodology, microphones are distributed upstream, downstream, and along the leading and trailing edges, enabling the study of the directivity of the acoustic field.

In the present work:

‐ One of the Brüel & Kjær 2250 units was used as a calibration reference and for spectral verification.
‐ The Casella CEL 63-C sound level meter was placed outside the test bench to record potential external interferences.
‐ The NoisePen devices were alternated between upstream and downstream positions related to the support frame to define the acoustic baseline and to assess the directivity of the aerodynamic noise.

### Acoustic characterization stages

Before addressing the acoustic characterization of the test bench, the operating condition of the flow was determined through pressure measurements in the square section of the diffuser. Using a micro-manometer, the differential pressure was recorded for the two operating settings of the blower (minimum and maximum velocity). From these measurements, the corresponding mean velocities at different distance from the diffuser output could be linked to the pressure and then,the “minimum velocity” and “maximum velocity” conditions used in this study could be set.

The standard deviations obtained (σ = 4.9 Pa for the minimum-velocity condition and σ = 1.6 Pa for the maximum-velocity condition) represent relative variations below 1% with respect to the mean pressure, confirming the remarkable steadiness of the blower’s operating regime during the measurements. This steadiness is relevant, as it ensures that the flow conditions used in the acoustic characterization are both representative and reproducible. Table 5 presents the average pressure values and the corresponding derived velocities for both configurations. Additionally, a fourth column was included reporting the mean incident velocity measured at 1 m from the diffuser, corresponding to the effective flow velocity impinging on a 0.20 × 0.20 m sample. This parameter is essential for defining the actual aerodynamic excitation acting on the test element.

**Table 5:** Operating conditions used for the acoustic characterization.

Test condition (Ref.)	Mean pressure in diffuser (Pa)	Std. dev. (σ) [Pa]	Reference velocity in diffuser	Mean incident velocity at 1 m (m/s)
**Minimum-velocity condition**	523.2	4.9	29.5	9.0
**Maximum-velocity condition**	683.4	1.6	33.7	12.0

### First stage – empty bench

**Fig. 10:**
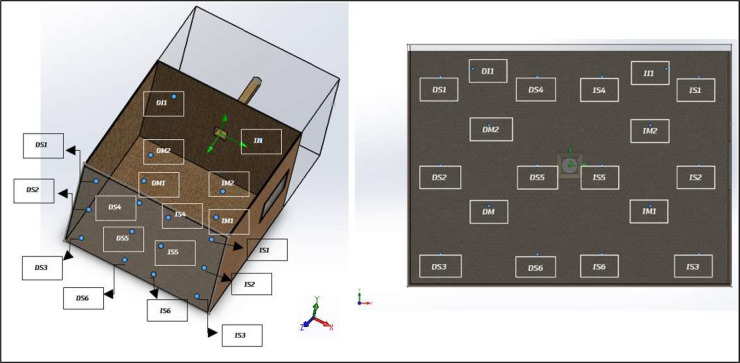
First stage of the acoustic characterization protocol, including baseline definition and measurement point layout within the test bench. This configuration establishes the reference acoustic conditions used for subsequent flow–structure interaction tests and residual energy analysis.

A complete sweep was performed with the bench empty, without the support frame or sample, in order to identify potential resonant modes of the chamber or tonal components from the blower that could interfere with future tests, as well as to define an acoustic baseline. Eighteen measurement points were established throughout the working volume (Fig. 10 and Table 6), where sound level meters and dosimeters were alternated to cover all positions. Measurements were carried out with the blower operating at minimum and maximum speed, thereby obtaining the acoustic baseline of the chamber.

**Table 6:** Coordinates of the measurement points for the acoustic characterization. Stage 1.

Reference Point	X [m]	Y [m]	Z [m]	Reference Point	X [m]	Y [m]	Z [m]
**DS1**	-0.8	0.55	2.0	**II1**	0.6	0.6	0.3
**DS2**	-0.8	0	2.0	**IS1**	0.8	0.55	2.0
**DS3**	-0.8	-0.55	2.0	**IS2**	0.8	0	2.0
**DS4**	-0.2	0.55	2.0	**IS3**	0.8	-0.55	2.0
**DS5**	-0.2	0	2.0	**IS4**	0.2	0.55	2.0
**DS6**	-0.2	-0.55	2.0	**IS5**	0.2	0	2.0
**DM2**	-0.5	-0.25	1.0	**IS6**	0.2	-0.55	2.0
**DM1**	-0.5	0.25	1.0	**IM1**	0.5	-0.25	1.0
**DI1**	-0.6	0.6	0.3	**IM2**	0.5	0.25	1.0

**Fig. 11:**
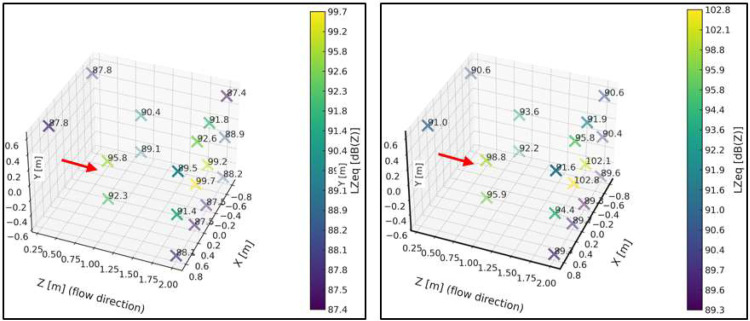
Spatial sound pressure level maps obtained within the working area of the test bench for two flow conditions. The left panel corresponds to the minimum-velocity condition, while the right panel corresponds to the maximum-velocity condition. The comparison illustrates the effect of flow velocity on the measured acoustic field under identical measurement geometry.

Fig. 11 presents a sound pressure map inside the test bench, where the sound pressure level is expressed in linear scale (dBZ) for both the minimum and maximum system velocities.

**Fig. 12:**
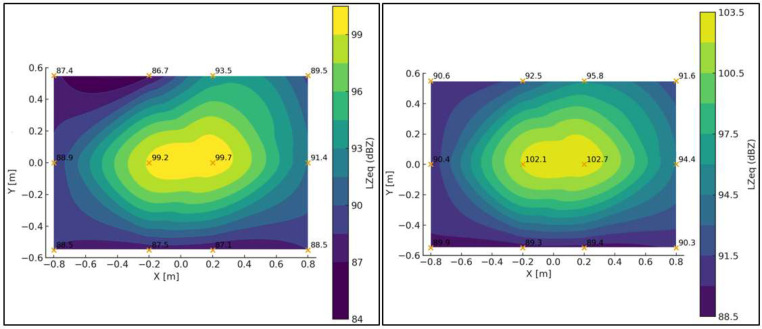
Sound pressure level maps obtained on the plane z=2m within the test bench. The left panel corresponds to the minimum-velocity condition, while the right panel corresponds to the maximum-velocity condition. The maps illustrate the spatial distribution of the acoustic field on a fixed measurement plane, enabling direct comparison between flow conditions.

For the plane z = 2 m, (Fig. 12) presents the unweighted (dBZ) sound pressure map for both operating conditions. The regions of higher sound pressure do not correspond to noise generated by the free turbulent flow—which does not radiate sound by itself—but rather to the unavoidable mechanical and aeroacoustic background of the test bench, mainly associated with the blower, minor interactions of the internal flow with structural elements, and the residual turbulence striking the walls. The purpose of this representation is therefore not to characterize a “flow-generated noise field,” but to identify steady background-noise patterns within the chamber. This allows delimiting areas without direct flow impingement or local interactions where microphones can be placed reliably, and provides the reference baseline required for the subsequent background-noise subtraction procedure.

### Second stage – bench with support frame

**Fig. 13:**
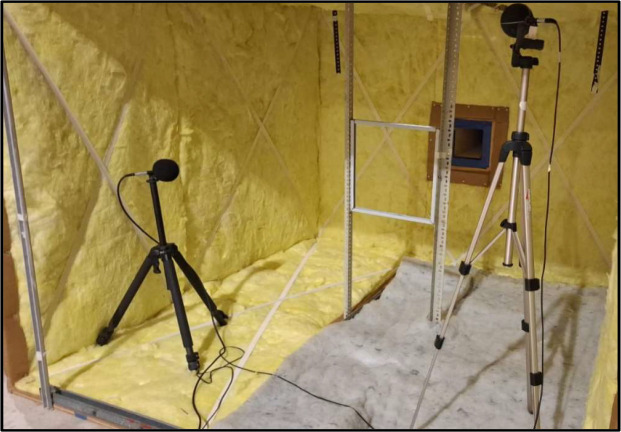
Second stage of the acoustic characterization protocol, showing the baseline measurement with the support frame installed at 50 cm from the diffuser and no test sample present. This configuration isolates the acoustic contribution of the support structure and establishes a corrected baseline for subsequent flow–structure interaction tests.

Keeping the same sensor distribution, the support frame (without sample) was placed 50 cm from the diffuser output (Fig. 13), with the objective of evaluating the potential acoustic impact of the structural support and its interaction with the flow.

**Fig. 14:**
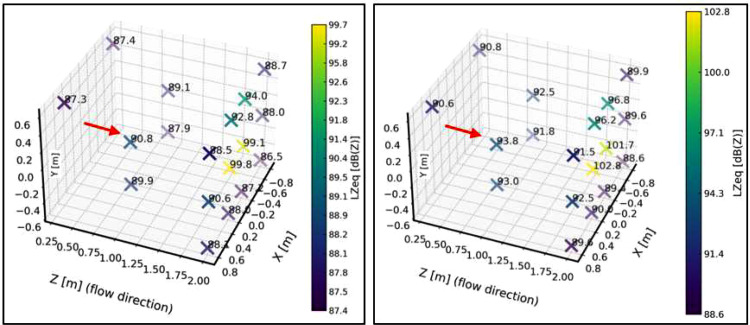
Acoustic results obtained during the second stage of the characterization protocol, corresponding to baseline measurements with the support frame installed at 50 cm from the diffuser and no test sample present. The left panel shows the low-speed operating condition, while the right panel shows the high-speed operating condition. These results quantify the acoustic contribution of the support structure and provide a reference for subsequent residual energy analysis.

In this stage, the sound pressure field around the support frame was again determined and expressed in decibels unweighted (dBZ). Regarding equivalent levels, no significant differences were observed compared to the condition without the frame, except at point IM2 (see Fig. 10), located in the central region. A decrease of approximately 5 dB was observed relative to the baseline condition without the frame. This localized behavior may be linked to a local interference effect or a slight modification of the acoustic field. Apart from this isolated case, the differences recorded at the remaining points remained below 2 dB, indicating that the overall influence of the support frame on the measured sound pressure levels is not significant. Fig. 14 shows the sound pressure map inside the test bench for this second characterization stage and for both selected velocities.

Under the maximum velocity condition, differences greater than 2 dB were observed at a few isolated points, particularly IM2 (–3.5 dB) and IM1 (–5 dB). These localized behaviors coincide with those identified under the minimum velocity condition and suggest local variations in the acoustic field associated with the presence of the support frame. Nevertheless, most points exhibited differences below 2 dB, supporting the conclusion that the frame does not introduce significant alterations in the overall distribution of sound levels. This characterization is essential for understanding the acoustic behavior of the test bench in the presence of the support frame, since when studying an obstacle, any potential modifications to the flow or the acoustic field caused by the frame must be considered to be avoided.

### Third stage – test according to ISO 3744:2010 [2]

In the context of aeroacoustic testing, the sound source is not a conventional standalone emitter but the combined flow–obstacle interaction, which radiates acoustic energy into the surrounding field. The concept of acoustic power remains well defined as long as the radiating region is enclosed by a control volume and the sound pressure is measured on a surface that surrounds that volume. In this work, the ISO 3744:2010 framework is not used for certifying the absolute sound power of a machine, but rather as a geometric and spatial sampling scheme, that ensures uniform coverage of the acoustic field around the source region. Under this interpretation, the obstacle immersed in the flow is treated as an equivalent acoustic source whose radiated power can be estimated from the pressure field measured on the control surface.

The methodology defined in [2] for determining sound power levels through sound pressure measurements in free-field conditions over a reflecting plane assumes that the source under test is placed on a rigid, reflective surface. However, due to the nature of the test bench—where the element under study (including the support frame) is suspended within the flow and not resting on a rigid surface—the control volume is not in direct contact with any physical reflecting plane.

The parallelepiped microphone arrangement defined in ISO 3744 was selected because the aeroacoustic source under investigation is not a compact or point-like emitter, but an extended flow–obstacle interaction region embedded within a rectangular test bench. This configuration allows a consistent definition of a control volume fully enclosing the radiating region and provides uniform spatial sampling under the geometric and operational constraints imposed by the experimental facility. Hemispherical or spherical arrangements, such as those defined in ISO 3744 and ISO 3745, are primarily intended for compact sources in free-field conditions and require physical configurations that are not compatible with an active flow environment or with the microphone velocity limitations present in the test bench. For these reasons, the parallelepiped arrangement was considered the most appropriate and robust option for the present experimental setup.

To reasonably comply with the principles of the standard, the lower face of the control volume was considered as an equivalent reflecting plane. This allows maintaining the microphone layout geometry proposed by [2], while adapting the position of the points so that they surround the control volume symmetrically and at equal distances, ensuring that the spatial relationships prescribed by the standard are preserved.

**Fig. 15:**
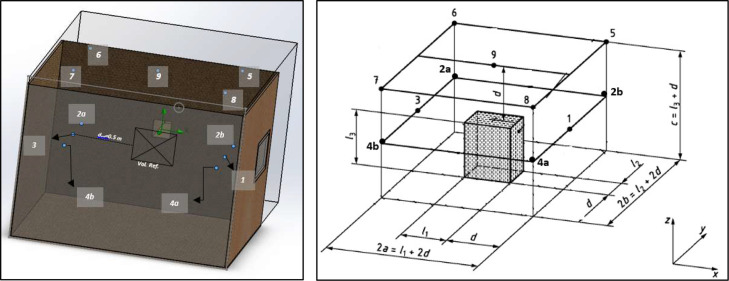
Control volume and microphone layout adapted from ISO 3744:2010 to the experimental test bench configuration. An imaginary reflecting plane is assumed below the control volume, while the remaining microphone positions are distributed around the other faces following the spatial coverage criteria of the standard. Measurement points 2 and 4 were divided into two positions (2-a/2-b and 4-a/4-b) to avoid direct exposure to the wind flow, preventing microphone velocities exceeding 5 m/s [2] .

In this context, the adaptation consists of assuming an imaginary reflecting plane located below the control volume, while the remaining microphone positions are distributed around the other faces, following the same logic of spatial coverage and acoustic representativeness defined in the standard. This adjustment preserves comparability with [2] criteria while respecting the experimental configuration and the actual operating conditions of the test bench. Figure shows the control volume with the adjusted measurement points adapted to the test bench. Both point 2 and point 4 were divided into two points—2-a and 2-b, and 4-a and 4-b, respectively (Fig. 15). This modification was implemented to avoid direct exposure to the wind flow, which would otherwise subject the microphones to velocities exceeding 5 m/s.

**Fig. 16:**
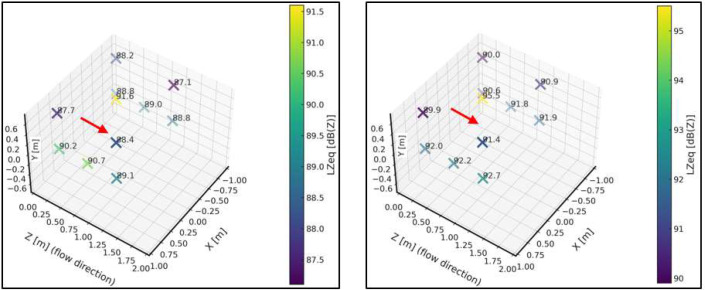
Acoustic characterization results obtained inside the test bench following the ISO 3744:2010 [2] measurement procedure adapted to the experimental configuration. The left panel corresponds to the low-speed operating condition, while the right panel corresponds to the high-speed operating condition. The comparison illustrates the influence of flow velocity on the measured acoustic field under identical measurement geometry.

Fig. 16 presents a sound pressure map inside the test bench, specifically within the volume defined by the [2]. The unweighted sound pressure level (dBZ) is shown for both the minimum and maximum operating velocities of the system.

### Integrated summary of the internal acoustic characterization of the test bench

Three measurement campaigns were carried out to establish the acoustic baseline of the bench: (i) empty bench, (ii) bench with the sample support frame installed 0.50 m downstream of the diffuser, and (iii) ISO 3744:2010 [2] application in two measurement planes (0.75 m and 1.00 m) surrounding a control volume. The results show a steady and homogeneous sound field (variations < 2–3 dB in regions outside the direct airflow) and a flat response up to 8 kHz.

A detailed comparison of the baseline measurements with and without the support frame shows that the acoustic field inside the bench remains globally steady. For the minimum-velocity condition, the absolute differences across the 18 measurement points remain below 2.4 dB, except at two specific locations: IM2 (−5.0 dB) and, to a lesser extent, IM1 (−2.4 dB). Both points are located near the geometric center of the test chamber, in the region most directly influenced by the structural presence of the frame. For the maximum-velocity condition, the same two points again exhibit the largest deviations: IM2 (−5.0 dB) and IM1 (−2.9 dB). A notable positive deviation also appears at DS4 (+4.9 dB), likely associated with a local modification of the flow field. Apart from these localized effects, all remaining points show differences below ±2 dB, confirming that the support frame does not induce significant changes in the overall distribution of sound pressure inside the test bench.

These localized deviations will be considered in future obstacle tests; however, they do not compromise the stability or representativeness of the baseline acoustic field used for the aeroacoustic analysis.

**Fig. 17:**
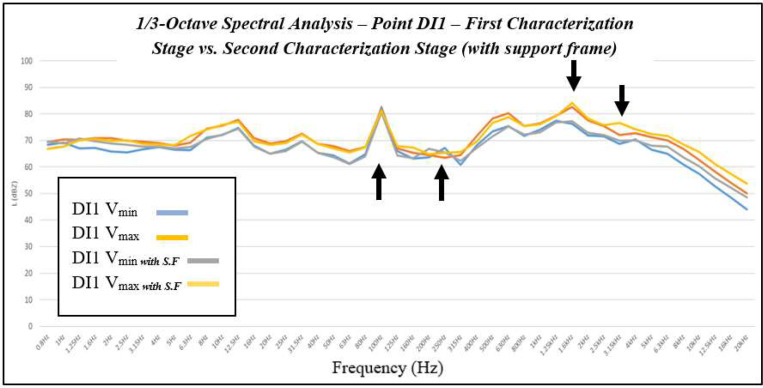
Third-octave band spectral comparison of the background noise measured at point DI1 during the first and second stages of the acoustic characterization protocol. The comparison evaluates the influence of the support structure on the baseline acoustic response of the test bench, serving as a reference for subsequent residual energy analysis.

Three main components of the background noise were identified (see Fig. 17):

(a) 100 Hz: electromechanical tone of the blower — persistent and independent of microphone position; (b) 250–315 Hz: room modes (harmonics of the natural frequencies computed using Eq. 1); (c) 1.6 kHz, and occasionally 3.15 kHz: edge-interaction effects near the diffuser outlet (see Fig. 16). The incorporation of the support frame did not generate new dominant tones; instead, it produced localized positional variations (1.6–3.15 kHz) associated with near-field flow interactions.

At points immersed in the flow (IS5/DS5), no significant differences were observed, and these locations are therefore proposed as reference stations for detecting flow-induced tones (see Fig. 10). These are strategic positions, as they are located within the flow, downstream of the interaction with the obstacle under study. However, the operating velocity must be verified to avoid microphone interference or saturation.

These in-flow measurement points are not intended for absolute sound power estimation, but for relative spectral analysis aimed at identifying flow–obstacle interaction mechanisms. Their use is consistent with the subtraction-based methodology adopted in this work, where baseline contributions are explicitly characterized and removed. Although the signal-to-noise ratio may be lower than at off-flow locations, these points provide higher sensitivity to local aeroacoustic phenomena under controlled and repeatable conditions.

The ISO 3744:2010 [2] estimation yielded consistent increases of approximately +2.7 dB and ≈ +3 dB(A) when transitioning from V_min_ to V_max_, evidencing the global effect of flow velocity on the acoustic field. This increase is not interpreted in terms of classical jet-noise scaling laws (e.g., U⁶ or U⁸), which are not directly applicable to the present experimental configuration. The test bench is dominated by background noise sources and localized flow–structure interaction mechanisms rather than free jet radiation. Consequently, the objective of this analysis is not to derive absolute aeroacoustic scaling relationships, but to verify the sensitivity, repeatability, and robustness of the measurement protocol under different flow conditions.

### Vibration measurement on the support frame

Prior to the measurements using the hot-wire anemometry technique, vibration recordings were carried out on the support frame in order to assess its structural stiffness and verify the absence of dynamic interferences during the experiments. Any undesired vibration could directly affect the hot-wire measurements; therefore, the structure was adjusted to simulate a typical test condition with an obstacle installed.

The support frame was positioned 50 cm downstream of the diffuser and tested under two blower operating conditions: minimum and maximum velocity. Measurements were performed using a Brüel & Kjær 2250 sound level meter (Class 1 [15],) and a Brüel & Kjær Type 4534-B-002 accelerometer, capable of recording from 0.8 Hz to 20 kHz, in both octave bands (OB) and third-octave bands (TOB).

The following non-interference criterion was adopted:

$$V_{vib}<min\left(0.05\sigma_{u},0.01U\right)in\;\;the\;\;range\;\;20-500\;\;Hz\;\;\left(extended\;\;to\;\;2\;\;kHz\right)$$

Where σᵤ is the standard deviation of the fluctuating component of the flow velocity, that is, a measure of the turbulence intensity, and *U* is the mean flow velocity. In addition, a conservative absolute limit of 0.10 m/s is imposed. In this way, the motion of the support frame remains below 5% of the turbulence level or 1% of the local mean velocity, whichever is more restrictive.

**Fig. 18:**
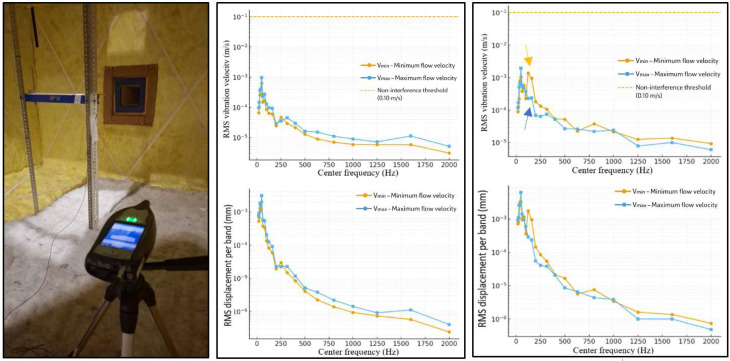
Vibration measurements performed on the support frame to assess structural stiffness and verify the absence of dynamic interference during the experiments. Vibrational velocity and displacement are shown for the central and lateral zones under minimum and maximum blower operating conditions. The measured vibration levels remain well below the adopted non-interference criteria, confirming that frame vibrations do not affect the hot-wire anemometry measurements.

As shown in Fig. 18, the vibrations measured at the centre of the frame were very low. The total RMS velocity between 20–500 Hz was 7.9 × 10⁻⁴ m/s at minimum blower speed and 1.2 × 10⁻³ m/s at maximum speed. Peak values at 50 Hz reached 6.3 × 10⁻⁴ m/s and 9.7 × 10⁻⁴ m/s, respectively. These correspond to RMS displacements between 2 µm and3 µm, i.e., two orders of magnitude below the conservative limit of 0.10 m/s [18]. Thus, frame vibrations do not affect the hot-wire measurements.

The procedure was then repeated by placing the accelerometer on one of the vertical lateral members of the frame. Under both operating conditions, the vibration levels remained below the adopted threshold (V_vib_ < 0.10 m/s). For reference, a velocity of 0.10 m/s at 50 Hz corresponds to a displacement amplitude of 0.32 mm, whereas the maximum measured value was only 0.006 mm.

An interesting result is that the vertical structure of the frame—1.50 m in length and rigidly connected to the top and bottom of the bench—exhibits favourable conditions for developing structural vibration modes between 100 Hz and 150 Hz. The coincidence with the 125 Hz component suggests the possible occurrence of a mechanical resonance of the first flexural mode, which may introduce a secondary vibro-acoustic coupling contribution. This effect is unrelated to the primary aeroacoustic phenomenon but will be taken into account in the spectral analysis of the obstacle tests.

### Example of aeroacoustic analysis – slatted panel case study

**Fig. 19:**
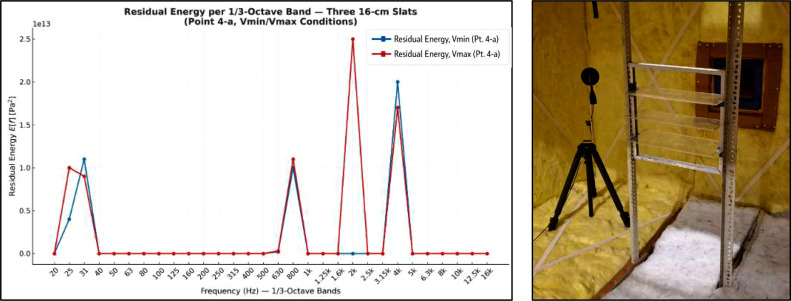
Example of a residual energy spectrum obtained using the proposed experimental methodology, shown for a three-slat configuration. This illustrative result demonstrates the capability of the test bench and the associated processing procedure to isolate aeroacoustic phenomena generated by flow–structure interaction, without constituting an exhaustive analysis of the specific configuration.

It should be noted that the objective of the present article is to document the design, construction, and acoustic characterization of a dedicated aeroacoustic test bench. The analytical procedures used to extract and interpret flow-induced tonal components, such as the subtraction-based residual energy method, are not the primary focus of this work and are therefore only introduced here in a simplified and illustrative manner. As an illustrative example, one of the preliminary tests performed inside the test bench involved the aeroacoustic characterization of a set of three acrylic slats spaced 10 cm apart, each measuring 16 cm in width, 40 cm in length, and 4 mm in thickness. Fig. 19 presents the residual energy spectrum (Pa² per band) obtained for this configuration, where a distinct tonal component associated with the interaction between the incoming flow and the slats can be observed. This result is consistent with the expected physical mechanisms for this type of geometry [17] and demonstrates the capability of the test bench to reliably detect aeroacoustic tones under controlled flow conditions.

In this context, the residual energy spectrum refers to the spectral difference obtained by subtracting a previously characterized baseline condition (test bench without obstacle) from the spectrum measured with the obstacle installed under identical flow conditions. This procedure highlights frequency bands where additional acoustic energy is introduced by the flow–obstacle interaction.

The residual energy spectrum shown in Fig. 19 is provided as an illustrative example to demonstrate that the test bench and its characterization protocol are capable of isolating flow-induced tonal components under controlled conditions. A full description and validation of the subtraction-based method is beyond the scope of this article and is addressed in separate doctoral research and ongoing publications.

## Method validation

The experimental characterization of the test bench —including three stages of acoustic verification and a complementary vibration assessment of the support frame— enabled a comprehensive validation of the system as a controlled environment for aeroacoustic studies.

The internal sound field was found to be steady and homogeneous, with variations below 3 dB at specific, identifiable frequencies. The volumetric and structural resonances identified (86–286 Hz and 250–315 Hz) were fully characterized as part of the acoustic baseline. These resonances will be considered during the analysis of future aeroacoustic measurements to avoid potential overlap or misinterpretation of spectral components. The composite material used in the construction of the bench provides a transmission loss of approximately 30 dBA and a reverberation time below 0.3 s, ensuring adequate isolation and internal damping.

Vibration measurements on the support frame showed RMS velocities between 7.9 × 10⁻⁴ m/s and 1.2 × 10⁻³ m/s, two orders of magnitude below the conservative interference threshold (V_vib_ < 0.10 m/s). Consequently, structural vibrations are acoustically negligible, and the frame behaves as a rigid body within the frequency range of interest.

As an operational criterion, a difference of Δ ≥ 3 dB relative to the acoustic baseline is attributed to the aeroacoustic response of the tested element. For smaller differences, a background-noise subtraction procedure—developed and documented as part of the doctoral research of the author—is applied. This procedure removes acoustic components identified during the test bench characterization. Filtering out these contributions, the resulting residual spectra isolate the acoustic signature associated exclusively with the flow–obstacle interaction. This subtraction procedure is mentioned here only for completeness; the present article is fully self-contained with respect to the design, construction, and validation of the test bench and does not rely on unpublished results.

With these acoustic and vibrational analysis, the test bench is fully characterized and operational for the detection, analysis, and attribution of aeroacoustic phenomena under controlled flow conditions (see Fig. 19). This analysis confirms that the proposed method can be reliably reproduced in other laboratory environments with comparable flow-control capabilities.

## Limitations

Not applicable.

## Ethics statements

This research complies with the ethical requirements of MethodsX. The study does not involve human participants, animal subjects, or any procedures requiring institutional ethical approval.

## CRediT authorship contribution statement

**Pablo Gianoli Kovar:** Investigation, Conceptualization, Methodology, Validation, Writing – original draft. **José Cataldo Ottieri:** Supervision, Validation, Writing – review & editing.

## Declaration of competing interest

The authors declare that they have no known competing financial interests or personal relationships that could have appeared to influence the work reported in this paper.

## Data Availability

Data will be made available on request.
